# Supplementary material for: Enhancing the understanding of environmental microbiomes through topic modeling: a quantitative and qualitative analysis
Source: Environ Microbiome. 2026 Jul 15;21:90. doi: 10.1186/s40793-026-00927-2 (PMC13374223; doi:10.1186/s40793-026-00927-2)
Supplement: Supplementary file 1 — Supplementary Material 1. [file 40793_2026_927_MOESM1_ESM.pdf]

Table 1. Random Forest Performances when predicting several target variables with the full microbiome (*none*, *fractions*, *clr*).

**Target**	R^2/Accuracy (***none***)	R^2/Accuracy (***fractions***)	R^2/Accuracy (***clr***)
Salinity	0.91	0.92	0.89
Chlorophyll a [mg/m^3]	0.57	0.49	0.57
Temperature [°C]	-1.3	-0.9	-0.6
Phosphate [μmol/l]	0.01	-0.00	-0.00
Ammonium [μmol/l]	-0.34	-0.89	-0.54
Nitrate [μmol/l]	0.09	0.12	-0.03
Nitrite [μmol/l]	0	0	0
Location ID	0.41	0.38	0.34
Calendar week	0	0	0

Table 2: Performance metrics (R^2, MAE, RSME) when predicting chlorophyll a with Random Forest based on the DRM approaches across $k \in \{2, 3, 4, \ldots, 20\}$.

k	Preprocession	Topic Model	Clustering Method	R^2	Mean Absolute error	Root Mean Squared Error
2	fractions	none	pcoa	0.4691931291545056	2.2271461187214605	4.603642864685615
2	none	none	pca	0.1972605096559211	2.8921004566210047	5.661358687786954
2	fractions	none	pca	0.0734250963868822	3.089383561643835	6.082381041370237
2	clr	none	pca	-0.2781110580545907	3.817762557077626	7.143608632353639
2	clr	lda	none	-2.982376686515864	6.095844748858448	12.60969336132591
2	none	nnmf	none	0.1212428851519551	2.899246575342465	5.923355309945022
2	fractions	nnmf	none	0.2392761231558812	2.679634703196347	5.511209001438273
2	clr	nnmf	none	-1.098528008124541	5.274771689497718	9.153574955987734
2	none	lda	none	-0.7769388591479114	3.975568130753062	8.423054285578184
2	fractions	lda	none	-2.8462401774677937	5.658219178082192	12.392289693015428
3	clr	lda	none	-0.0554793848785781	2.956164383561644	6.49169690967803
3	fractions	none	pcoa	0.2846597037033557	2.935730593607306	5.344286134951743
3	none	none	pca	0.2422896648666093	2.805593607305936	5.500282080769725
3	fractions	none	pca	0.1847435290366953	2.9018949771689497	5.705326258584601
3	clr	none	pca	0.3346670361562431	2.804269406392694	5.154100442352963
3	none	nnmf	none	0.2783217847670344	2.6560273972602744	5.367909127567873
3	fractions	nnmf	none	0.349688433025804	2.5864155251141554	5.09558554134188
3	clr	nnmf	none	0.0608201241397243	3.2902739726027392	6.123613140911449
3	none	lda	none	0.3434621104379826	2.538904109589041	5.119920937959775
3	fractions	lda	none	-0.9164920341587904	5.746164383561643	8.747558720102646
4	fractions	none	pcoa	0.4451426178849561	2.383401826484018	4.706781540049785
4	none	none	pca	0.3003144538807762	2.7277625570776256	5.285484559807499
4	fractions	none	pca	0.27614841786083	2.791438356164384	5.375985902997119
4	clr	none	pca	0.2152290027776342	3.017602739726028	5.59763826176621
4	fractions	nnmf	none	-0.1741629420832728	3.254954337899544	6.846956078013542
4	clr	lda	none	0.5019643952888317	2.3397945205479456	4.459267784971226
4	fractions	lda	none	-1.085673676707195	6.19607305936073	9.125497221706825
4	clr	nnmf	none	0.1208378880737192	3.0798858447488584	5.924720115540361
4	none	nnmf	none	0.482605791124417	2.3528995433789954	4.545107284576515
4	none	lda	none	0.4229731478130636	2.385981735159817	4.799890932931344
5	none	none	pca	0.3335034629562613	2.662716894977169	5.15860536974196
5	clr	lda	none	0.4761515011444227	2.2936757990867584	4.573368636457323
5	fractions	lda	none	-1.1438165979699615	6.0492694063926935	9.25181993697017
5	none	lda	none	0.5415363197992207	1.9993150684931509	4.278443738313945
5	clr	nnmf	none	0.4697451639712805	2.213767123287671	4.601248366441477
5	none	nnmf	none	0.4415333142987845	2.372716894977169	4.7220653470226885
5	clr	none	pca	0.2999605660545845	2.9154794520547944	5.2868210403160765
5	fractions	none	pca	0.2464849236189769	2.7867808219178083	5.48503407997138
5	fractions	nnmf	none	0.238687165935834	2.700296803652968	5.513341995046009
5	fractions	none	pcoa	0.4708040998694996	2.592328767123288	4.596651650135571
6	fractions	lda	none	-0.5568351525035524	5.574155251141552	7.88414625057505
6	clr	lda	none	0.3909665934267358	2.6017123287671238	4.931214735450413
6	fractions	none	pcoa	-0.3977033569737731	4.214657534246576	7.470348478665106

6	none	none	pca	0.3181738666610268	2.683264840182648	5.21759275920546
6	fractions	none	pca	0.1883587223666132	2.919018264840183	5.692662284140746
6	clr	none	pca	0.2625511381563589	2.7057762557077623	5.426243918159295
6	none	nnmf	none	-0.0930974984059544	3.213607305936073	6.606368692855105
6	fractions	nnmf	none	0.3578816212422432	2.6490867579908675	5.063384496271009
6	clr	nnmf	none	0.4994308047932597	2.4124657534246574	4.470595917087707
6	none	lda	none	0.4757611312161901	2.5506164383561645	4.575072347657616
7	fractions	none	pcoa	0.3543790821274202	2.302716894977169	5.077175242936626
7	fractions	none	pca	0.1373584992002358	3.028584474885845	5.868789479470012
7	clr	none	pca	0.1259533935545728	3.115616438356164	5.90745813729015
7	none	none	pca	0.3406349419485424	2.586575342465753	5.13093273940087
7	none	nnmf	none	0.4082303015135208	2.5754109589041096	4.860822012712398
7	clr	lda	none	0.3015783128339856	2.825388127853881	5.280708752762914
7	fractions	lda	none	-1.2094043726197028	7.026666666666667	9.392278500650946
7	clr	nnmf	none	0.3919876634190437	2.6681278538812787	4.927079307488677
7	fractions	nnmf	none	0.2258432645112978	3.29662100456621	5.559654525911531
7	none	lda	none	0.5728053295953552	2.117694063926941	4.12996403955731
8	clr	lda	none	0.5818283998602567	2.281187214611872	4.086115369490366
8	fractions	lda	none	-4.303631581371521	9.36849315068493	14.551911045237087
8	none	lda	none	0.2727075111403025	2.870057077625571	5.388748418958808
8	clr	nnmf	none	0.5994298189916233	2.174086757990868	3.999195781026126
8	none	nnmf	none	0.4811884500237343	2.3036529680365296	4.551328422665069
8	clr	none	pca	0.2400947746638404	3.034657534246575	5.5082427648175045
8	fractions	none	pca	0.2401844074145446	2.62175799086758	5.50791789962787
8	none	none	pca	0.3139016981480872	2.781392694063927	5.233913362557503
8	fractions	none	pcoa	0.4442054109817597	2.3615525114155247	4.710754963778856
8	fractions	nnmf	none	0.4708301748515227	2.4340182648401822	4.5965384037124775
9	fractions	none	pcoa	0.4921324459160318	2.220616438356165	4.503068892661402
9	fractions	lda	none	0.0199075967432905	3.471780821917808	6.25556971917151
9	none	lda	none	0.4636630829232679	2.649429223744292	4.627561539200398
9	clr	nnmf	none	0.4808657263756182	2.5508219178082188	4.552743766025798
9	fractions	nnmf	none	0.4748777096816139	2.401963470319634	4.578925568376341
9	none	nnmf	none	0.5439971691004256	2.126849315068493	4.266945802798328
9	clr	none	pca	0.0567694443008365	3.346392694063927	6.136804494718135
9	fractions	none	pca	0.3004521823932772	2.5036529680365294	5.28496432771362
9	clr	lda	none	0.4191296325627082	2.6607534246575346	4.815850185969102
9	none	none	pca	-0.1760650033760866	3.582922374429224	6.852499627256649
10	clr	lda	none	0.3326359837571848	2.8231963470319634	5.16196137194763
10	clr	none	pca	-0.0297364986299824	3.569794520547945	6.412042768553047
10	fractions	lda	none	-0.6861013733012764	4.792406098515691	8.204936008928922
10	none	lda	none	0.4718056879618619	2.558219178082192	4.592299639803043
10	clr	nnmf	none	0.4264402970016276	2.650502283105023	4.785448786824931
10	fractions	nnmf	none	0.4489305694071717	2.3533561643835617	4.690687680206604
10	none	nnmf	none	0.516962530529693	2.2025342465753424	4.3916100219025465
10	fractions	none	pca	0.3777528789951011	2.512077625570776	4.984422011165199
10	none	none	pca	0.1082084419399259	3.295296803652968	5.967123627306681
10	fractions	none	pcoa	0.5457222464527591	2.22689497716895	4.258867139870472
11	none	lda	none	0.4512989668861514	2.7150000000000003	4.680596959741714
11	fractions	none	pca	0.3702481846680232	2.507853881278539	5.014389568005864
11	clr	none	pca	-0.236858789653352	3.7262100456621	7.027379656755614
11	none	nnmf	none	0.5764206762983051	2.1513470319634704	4.126988492625687
11	clr	lda	none	0.5571835734193099	2.313744292237443	4.20479879146581
11	fractions	nnmf	none	0.4656064353224828	2.4683333333333337	4.619170224363229
11	clr	nnmf	none	0.460596785119526	2.479223744292238	4.6407708283515285
11	fractions	lda	none	0.2080416405815083	3.043006052116385	5.623212955501695
11	none	none	pca	0.0489085748684423	3.2184246575342463	6.16232345510172
11	fractions	none	pcoa	0.0829611976412825	3.1595205479452053	6.051000841779367
12	clr	none	pca	0.0630462414207448	3.386301369863013	6.116351502960124
12	fractions	none	pcoa	0.3867215177350297	2.802899543378995	4.9483706326232495
12	fractions	none	pca	0.3582987523362363	2.5420547945205483	5.061739598453982

12	none	nnmf	none	0.576483480283184	2.127511415525114	4.112146086144065
12	clr	nnmf	none	0.4308173944444182	2.9152511415525115	4.7671538372243205
12	fractions	nnmf	none	0.2396072182455942	2.88324200913242	5.510009530194212
12	clr	lda	none	0.3604067888324379	2.865388127853881	5.05341866291013
12	none	none	pca	0.1219995571138297	3.2071232876712323	5.920804547020469
12	fractions	lda	none	-3.322821056436248	8.782428127491483	13.137628785687571
12	none	lda	none	0.4341688873640361	2.8856164383561644	4.753098004382974
13	fractions	none	pcoa	0.3330901230274176	3.258675799086758	5.160204723205091
13	none	none	pca	-0.1042501913637978	3.385570776255708	6.639985008510336
13	clr	none	pca	0.1298138861342318	3.2226027397260277	5.894397657461892
13	clr	lda	none	0.4908307896752847	2.480616438356164	4.508835845862157
13	fractions	none	pca	0.4007711183228291	2.518287671232877	4.891361106105297
13	none	nnmf	none	0.4523048950906529	2.385890410958904	4.6763045453555785
13	fractions	nnmf	none	0.3451865645067544	2.6856392694063924	5.113192557282963
13	clr	nnmf	none	0.4449381408493952	2.7060730593607305	4.707648735940753
13	none	lda	none	0.3601022318987706	2.9539041095890406	5.054621670146095
13	fractions	lda	none	-0.2225880377651716	3.973173515981735	6.986721440276424
14	fractions	none	pca	0.4296505332806944	2.342716894977169	4.772037822269324
14	none	none	pca	-0.2240653970700892	3.645022831050228	6.990941496944358
14	none	lda	none	0.2528084841605919	2.932214611872146	5.46197016071396
14	clr	none	pca	-0.1307342017944175	3.5987899543378994	6.719138939045076
14	fractions	none	pcoa	0.3017050852794788	2.9521004566210047	5.280229473012629
14	fractions	lda	none	-0.3251053047878516	3.9982891117961934	7.273753070559401
14	none	nnmf	none	0.4003425165240251	2.339931506849315	4.893110080072566
14	fractions	nnmf	none	0.3561633371089105	2.5802283105022834	5.070154681383072
14	clr	nnmf	none	0.2292752296287243	3.28148401826484	5.547317402609734
14	clr	lda	none	0.496056216072679	2.396141552511416	4.4856398728044535
15	clr	lda	none	0.426304386876031	2.9917351598173552	4.786015730787192
15	fractions	lda	none	-2.3588818223373598	8.018607704320035	11.580590884562222
15	none	lda	none	0.2739634144220781	3.192625570776256	5.3840937091114185
15	clr	nnmf	none	0.4136951717260967	2.8781050228310505	4.838325614182912
15	fractions	nnmf	none	0.5586804718061515	2.108493150684932	4.1976858160235935
15	none	nnmf	none	0.3623647340724691	2.385296803652968	5.0456778827828135
15	clr	none	pca	-0.170006138211191	3.747260273972602	6.834825441281247
15	fractions	none	pca	0.1114723835328435	3.232579908675799	5.956193833075696
15	none	none	pca	-0.0264594051820974	3.741552511415525	6.401831608626197
15	fractions	none	pcoa	0.4000082020889437	2.630684931506849	4.894473866435875
16	fractions	none	pcoa	0.1664408301256591	3.784520547945205	5.769013748874516
16	clr	none	pca	-0.6412927454869317	4.4527168949771685	8.09517761910951
16	fractions	none	pca	0.3842076378673158	2.5032191780821917	4.958502153605052
16	fractions	nnmf	none	0.4753257876621657	2.210867579908676	4.5769715915408185
16	none	nnmf	none	0.3815400128150297	2.374018264840183	4.9692307132270015
16	clr	lda	none	0.5362891528137449	2.4968036529680364	4.302857710276491
16	none	none	pca	-0.0374716935108638	3.605022831050229	6.436080765220272
16	none	lda	none	0.3073485405279228	3.0387442922374426	5.258849401909458
16	clr	nnmf	none	0.3356200081308074	3.0743607305936065	5.150407950327286
16	fractions	lda	none	0.1413004932533099	2.9986073059360727	5.855364885744139
17	clr	lda	none	0.5755083445739003	2.116872146118721	4.116877418671255
17	fractions	lda	none	-1.4276757630474737	5.889987214192476	9.845294202597776
17	none	lda	none	0.326645245201022	2.8947945205479453	5.185078344916533
17	clr	nnmf	none	0.2741963795191385	3.4685844748858443	5.383229836289701
17	none	nnmf	none	0.4003544789938478	2.347785388127854	4.893061273899703
17	clr	none	pca	-0.2343159871411049	3.965799086757991	7.020152302917964
17	fractions	none	pca	0.3360837940007488	2.682739726027397	5.148609955598292
17	none	none	pca	-0.0567177177967548	3.76351598173516	6.4955039596245125
17	fractions	none	pcoa	0.1657378177320531	2.6177853881278543	5.771445989765943
17	fractions	nnmf	none	0.3928923394948872	2.268173515981735	4.923412383471132
18	fractions	lda	none	0.0195010235754313	3.6053164090590943	6.256867088214808
18	fractions	none	pca	0.3120092491457951	2.7847260273972605	5.24112668269997
18	fractions	none	pcoa	0.3178881340345566	2.697990867579908	5.218685912028222

18	none	none	pca	-0.391337264476832	4.1901826484018265	7.453316536768064
18	clr	none	pca	-0.1597550620933618	4.141849315068493	6.804817709823456
18	clr	lda	none	0.4245700803524289	2.580136986301369	4.793244437481039
18	none	nnmf	none	0.3501717980050539	2.4451598173515983	5.09369146043063
18	fractions	nnmf	none	0.3941521042184635	2.31703196347032	4.918301624298769
18	clr	nnmf	none	0.3435655640727981	2.961735159817352	5.119517537556796
18	none	lda	none	0.3876446750262019	2.819703196347032	4.944644882466144
19	none	nnmf	none	0.3777810182465343	2.358127853881278	4.984309307156448
19	clr	lda	none	0.5907415434904193	2.1455022831050226	4.0423339419981295
19	fractions	lda	none	-1.5564351521669368	5.601565459219914	10.103009179241226
19	none	lda	none	0.4015390208252064	2.73337899543379	4.888225999752623
19	clr	nnmf	none	0.3686941386122482	2.95351598173516	5.020572790185649
19	fractions	nnmf	none	0.4026637045618685	2.2204337899543383	4.883630634427218
19	clr	none	pca	-0.5660826162869943	4.461347031963471	7.907527149269762
19	fractions	none	pca	0.3995387266121792	2.461735159817352	4.896388381050077
19	none	none	pca	-0.1096118709786577	3.785707762557078	6.656085689977914
19	fractions	none	pcoa	0.3046439987905619	2.9468493150684933	5.269106307386369
20	none	none	pca	-0.2900033427174446	3.874931506849314	7.176765816153449
20	fractions	none	pca	0.3955038232810549	2.570525114155251	4.912811901167773
20	clr	none	pca	-0.1916914942199203	4.12513698630137	6.897874316869234
20	fractions	nnmf	none	0.3914039154772031	2.3984018264840183	4.929443965420358
20	none	nnmf	none	0.3767087589474128	2.462762557077625	4.988602145878445
20	clr	nnmf	none	0.3793822051250718	2.8453424657534248	4.977891991174486
20	none	lda	none	0.3750616949678292	2.9601826484018265	4.995189055344166
20	fractions	lda	none	0.581546938727552	2.2237899543378994	4.087490270609677
20	clr	lda	none	0.4624562744296294	2.447579908675799	4.6327648381568105
20	fractions	none	pcoa	0.1428786758220497	3.378812785388128	5.849981696103746

Table 3. The approaches, where $R^2 > baseline$, their bootstrapped mean R^2, t-test p-values, and significance at $\alpha = 0.05$. The test evaluates whether the bootstrapped R^2 distributions differ from the full microbiome baseline.

**Approach**	R^2	Bootstrapped mean R^2	p-value	significant
clr + nnmf + k=8	0.6	0.57	0.02	yes
none + nnmf + k=12	0.58	0.51	< 0.01	yes
clr + lda + k=19	0.59	0.55	< 0.01	yes
fractions + lda + k=20	0.58	0.59	< 0.01	yes

Table 4. Bacterial identification numbers of the top 5 contributing ASVs across extracted topics.

Phylum	Class	Ordner	Family	Genus	ID
Proteobacteria	Alphaproteobacteria	Rhodobacterales	Rhodobacteraceae	*Pseudorhodobacter*	9129c25a-b415-46d9-8bce-618c0704cbfd
Proteobacteria	Alphaproteobacteria	Rhodobacterales	Rhodobacteraceae	*Pseudorhodobacter*	7fb18c69-c34c-4630-94ef-c4515c4066c8
Proteobacteria	Alphaproteobacteria	Rhodobacterales	Rhodobacteraceae	*Tabrizicola*	da897aba-58df-408a-a5b6-023b6da4b80c
Proteobacteria	Alphaproteobacteria	Rhodobacterales	Rhodobacteraceae	*Yoonia-Loktanella*	e6368140-273a-40c7-a909-d8dd48618e79
Proteobacteria	Alphaproteobacteria	Rhodobacterales	Rhodobacteraceae	*Yoonia-Loktanella*	6c8700a1-b831-47ad-a78d-91c850bf6459
Proteobacteria	Alphaproteobacteria	Rhodobacterales	Rhodobacteraceae	*Planktomarina*	307a04d-0ecb-45fd-95e3-519fc0d1ba32
Proteobacteria	Alphaproteobacteria	Rhodobacterales	Rhodobacteraceae	*Planktomarina*	f202e1c3-12fe-4a09-904d-86cd64146552
Proteobacteria	Alphaproteobacteria	Rhodobacterales	Rhodobacteraceae	*Lentibacter*	90fdc862-54ed-4ed3-b394-8314995dc79a
Proteobacteria	Alphaproteobacteria	Rhodobacterales	Rhodobacteraceae	*Seohaeicola*	4e7a02b2-0b3e-4f26-b715-5d0238df4b47
Proteobacteria	Alphaproteobacteria	Rhodobacterales	Rhodobacteraceae	*Marivita*	c5055337-5e3f-409a-a65c-52ee5ee92744
Proteobacteria	Alphaproteobacteria	Rhodobacterales	Rhodobacteraceae	*Rhodobacter*	05fab431-d352-4c80-954e-0107499d1b6c
Proteobacteria	Gammaproteobacteria	Burkholderiales	Burkholderiaceae	*Polynucleobacter*	fcecd815-81d7-4dda-8a51-761486ec0e05
Proteobacteria	Gammaproteobacteria	Burkholderiales	Comamonadaceae	*RS62 marine group*	ae897dbb-82f8-49ce-a62e-592e687bbb5f
Proteobacteria	Gammaproteobacteria	Pseudomonadales	SAR86 clade	SAR86 clade (unclassified)	6715e23a-6d57-446b-90a7-0f4359c6a3cd
Cyanobacteria	Cyanobacteriia	Synechococcales	Cyanobiaceae	*Cyanobium PCC-6307*	167cd9db-5f68-4258-b1a8-d50ba42d3f6f
Cyanobacteria	Cyanobacteriia	Synechococcales	Cyanobiaceae	*Cyanobium PCC-6307*	d4be031f-3dde-45ae-aadc-9e4b111a7d17
Cyanobacteria	Cyanobacteriia	Synechococcales	Cyanobiaceae	*Cyanobium PCC-6307*	c4e5479a-310f-43eb-8569-9a5df2af6873
Cyanobacteria	Cyanobacteriia	Synechococcales	Cyanobiaceae	*Cyanobium PCC-6307*	d5971909-dd7b-40f5-8351-6a992a9e25a2
Cyanobacteria	Cyanobacteriia	Synechococcales	Cyanobiaceae	*Synechococcus CC9902*	31c638bb-18d2-4f9c-a320-d454c56aa9b9
Bacteroidota	Bacteroidia	Flavobacteriales	Flavobacteriaceae	*NS3a marine group*	9fbd4c78-3fce-4db9-a574-c0b8b005040e
Bacteroidota	Bacteroidia	Flavobacteriales	Flavobacteriaceae	*NS3a marine group*	1d8af072-a47a-44dd-9a65-ac125b94bdec
Bacteroidota	Bacteroidia	Flavobacteriales	Flavobacteriaceae	*NS5 marine group*	1a86ec6b-4ce3-40ea-bd95-f5daf3c1d87d
Bacteroidota	Bacteroidia	Flavobacteriales	Flavobacteriaceae	*Polaribacter*	fd77a141-bf9a-43ce-89ea-fe48914b4eca
Bacteroidota	Bacteroidia	Flavobacteriales	Flavobacteriaceae	Flavobacteriaceae (unclassified)	d75b3c67-0803-4297-bc7c-c6a863521fa2
Bacteroidota	Bacteroidia	Flavobacteriales	Cryomorphaceae	Cryomorphaceae (unclassified)	865ea2ae-30d7-4a54-ad63-3c5b2ac3636c
Bacteroidota	Bacteroidia	Flavobacteriales	Crocinitomicaceae	Crocinitomicaceae (unclassified)	a92c9174-9b90-4da4-842b-20ff64afc329
Actinobacteriota	Acidimicrobiia	Microtrichales	Ilumatobacteraceae	*Ilumatobacter*	6a2396a5-2e01-4512-9981-0b79f530c193
Actinobacteriota	Acidimicrobiia	Microtrichales	Ilumatobacteraceae	Ilumatobacteraceae (unclassified)	49ad3683-413f-4e97-b61d-2536d67b98a9
Actinobacteriota	Actinobacteria	Corynebacteriales	Mycobacteriaceae	*Mycobacterium*	adbd1e81-ffce-460e-89e8-5ecf19c6bb65
Actinobacteriota	Actinobacteria	Corynebacteriales	Mycobacteriaceae	*Mycobacterium*	a6e3477b-3b31-43a5-9711-bda88ee53f66
Actinobacteriota	Actinobacteria	Micrococcales	Microbacteriaceae	*Candidatus* Limnoluna	381985e7-57c3-410d-adec-0af27163e4db
Actinobacteriota	Actinobacteria	Frankiales	Sporichthyaceae	Sporichthyaceae (unclassified)	9ef8efbe-4313-4521-856c-f9924d47c75f
Actinobacteriota	Actinobacteria	Micrococcales	Microbacteriaceae	*Candidatus* Aquiluna	da565a40-88bf-4491-8a27-cd67e928b714
Actinobacteriota	Actinobacteria	Micrococcales	Microbacteriaceae	*Candidatus* Aquiluna	6b70ca18-a760-4b45-954c-bc86f65afaea
Actinobacteriota	Actinobacteria	Micrococcales	Microbacteriaceae	*Candidatus* Aquiluna	608fed8c-61ae-4e37-b740-d9c1c6f4d785
Actinobacteriota	Acidimicrobiia	Microtrichales	Ilumatobacteraceae	*CL500-29 marine group*	d481a517-1f38-4b72-93ad-1c32e42bbbcd
Actinobacteriota	Acidimicrobiia	Microtrichales	Ilumatobacteraceae	*CL500-29 marine group*	0e355dc3-7810-4603-b37a-03639af58466
Actinobacteriota	Acidimicrobiia	Microtrichales	Ilumatobacteraceae	*CL500-29 marine group*	b7afcb6d-b3d5-4eb5-b346-6527f092b9fa
Actinobacteriota	Actinobacteria	Frankiales	Sporichthyaceae	*hgcI clade*	06802502-e084-41f4-9a57-98a020f96856
Actinobacteriota	Actinobacteria	Frankiales	Sporichthyaceae	*hgcI clade*	cc4e982f-16b2-419d-972f-b32e9c590c04
Actinobacteriota	Actinobacteria	Frankiales	Sporichthyaceae	*hgcI clade*	db72cb19-c152-4378-8845-484741bc81ab
Actinobacteriota	Actinobacteria	PeM15	PeM15 unclassified	PeM15 (unclassified)	92c9e165-c9b3-44b4-8c15-1e6a25d411e5
Actinobacteriota	Actinobacteria	PeM15	PeM15 (unclassified)	PeM15 (unclassified)	e730717f-2fa8-444e-9dcc-c407e31b1d9b
Actinobacteriota	Actinobacteria	Micrococcales	Microbacteriaceae	*ML602J-51*	a5045aa2-f32c-4e0a-a8c4-c0f7f51a22e8
Verrucomicrobiota	Verrucomicrobiae	Verrucomicrobiales	Rubritaleaceae	*Luteolibacter*	623f6a46-892a-4217-9faf-5b372e1221b3
Verrucomicrobiota	Verrucomicrobiae	Verrucomicrobiales	Rubritaleaceae	*Luteolibacter*	fbaaa28c-9fe5-401e-893d-98e6a3aee734
Planctomycetota	Planctomycetes	Planctomycetales	Rubinisphaeraceae	*Fuerstia*	8f15350d-04e5-448a-8267-5aff58f4d3a7

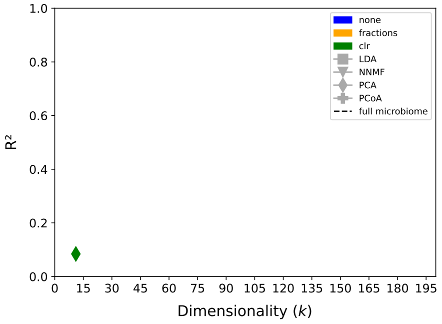

Fig. 1. RF performance (R^2) based on DMR generated topic or PCA/PCoA-component clusters across $k \in \{11, 21, \ldots, 191\}$ when predicting temperature. RF performance (Full microbiome)<0.15

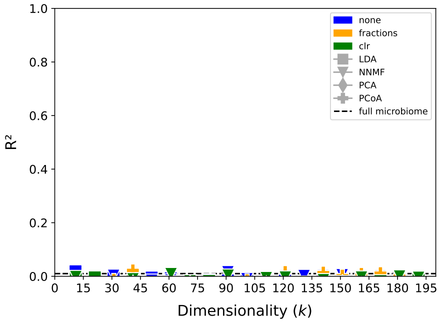

Fig. 2. RF performance (R^2) based on DMR generated topic or PCA/PCoA-component clusters across $k \in \{11, 21, \ldots, 191\}$ when predicting phosphate concentrations. RF performance (Full microbiome)<0.15

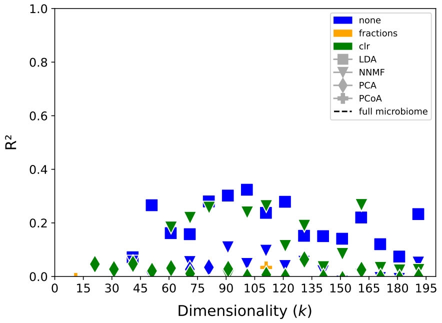

Fig. 3. RF performance (R^2) based on DMR generated topic or PCA/PCoA-component clusters across $k \in \{11, 21, \ldots, 191\}$ when predicting ammonium concentrations. RF performance (Full microbiome)<0.15

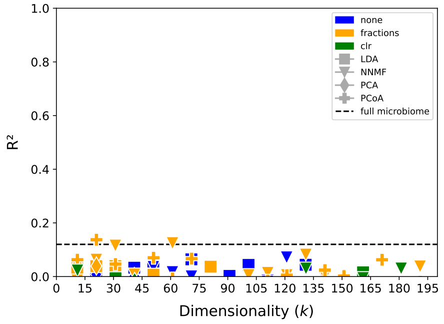

Fig. 4. RF performance (R^2) based on DMR generated topic or PCA/PCoA-component clusters across $k \in \{11, 21, \ldots, 191\}$ when predicting nitrate concentrations. RF performance (Full microbiome)<0.15

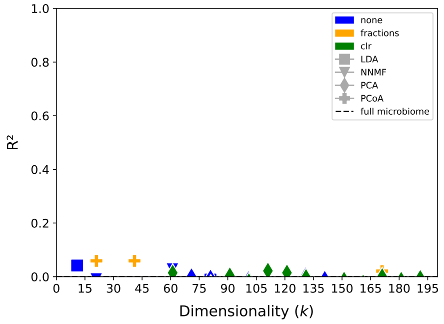

Fig. 5. RF performance (R^2) based on DMR generated topic or PCA/PCoA-component clusters across $k \in \{11, 21, \ldots, 191\}$ when predicting nitrite concentrations. RF performance (Full microbiome)<0.15

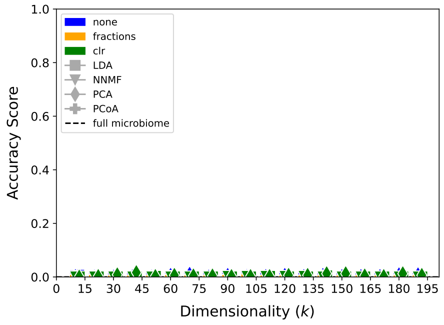

Fig. 6. RF performance (Accuracy) based on DMR generated topic or PCA/PCoA-component clusters across $k \in \{11, 21, \ldots, 191\}$ when predicting the calendar week. RF performance (Full microbiome)<0.1

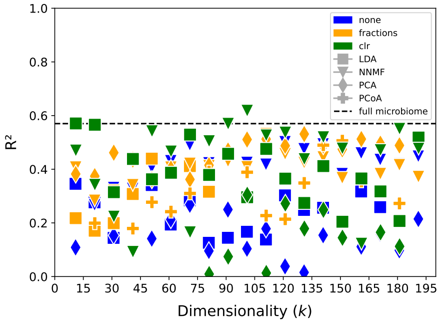

Fig. 7. RF performance (R^2) based on DMR generated topic or PCA/PCoA-component clusters across $k \in \{11, 21, \ldots, 191\}$ when predicting chlorophyll a.

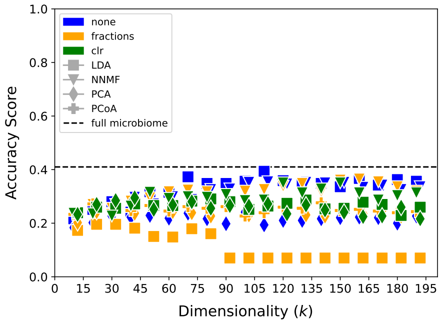

Fig. 8. RF performance (Accuracy) based on DMR generated topic or PCA/PCoA-component clusters across $k \in \{11, 21, \ldots, 191\}$ when predicting the location id.

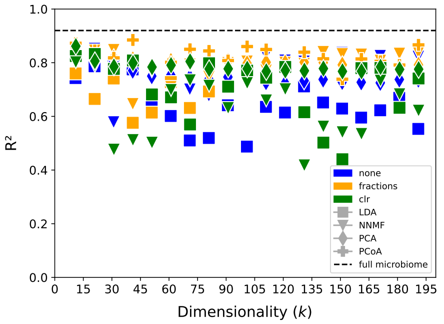

Fig. 9. RF performance (R^2) based on DMR generated topic or PCA/PCoA-component clusters across $k \in \{11, 21, \ldots, 191\}$ when predicting salinity.

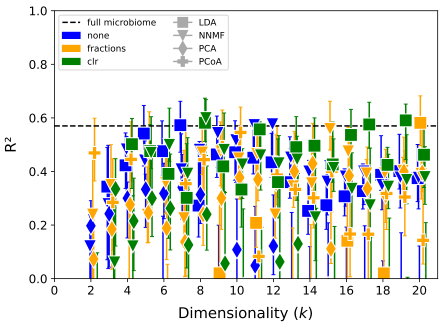

Fig. 10. RF performance (R^2) based on DMR generated topic or PCA/PCoA-component clusters across $k \in \{2, 3, 4, ..., 20\}$ when predicting chlorophyll a concentration, with 95% confidence intervals.

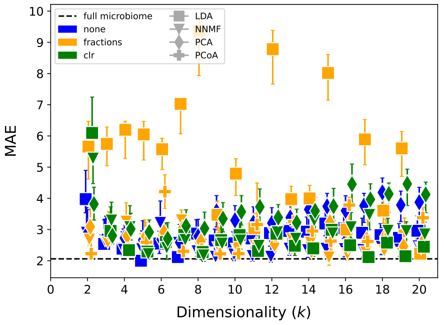

Fig. 11. RF performance (MAE) based on DMR generated topic or PCA/PCoA-component clusters across $k \in \{2, 3, 4, ..., 20\}$ when predicting chlorophyll a concentration, with 95% confidence intervals.

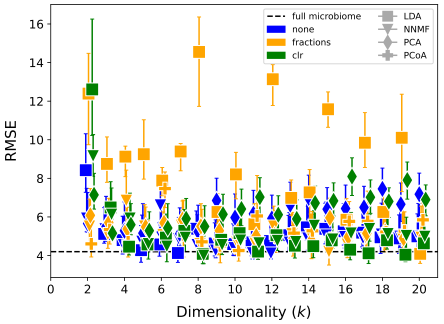

Fig. 12. RF performance (RMSE) based on DMR generated topic or PCA/PCoA-component clusters across $k \in \{2, 3, 4, ..., 20\}$ when predicting chlorophyll a concentration, with 95% confidence intervals.

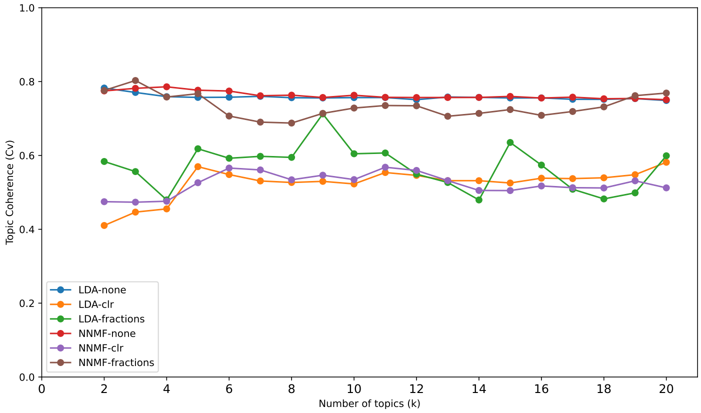

Fig. 13. The results of the topic coherence analysis using C_v as the coherence metric for LDA and NNMF topics based on various preprocessed microbiome data across $k \in \{2, 3, 4, \ldots, 20\}$. The NNMF model with unprocessed data (red) selected in our study shows that topic coherence is among the highest performing approaches here. Furthermore the analysis suggests that the choice of k does not influence topic coherence drastically across the k range considered.

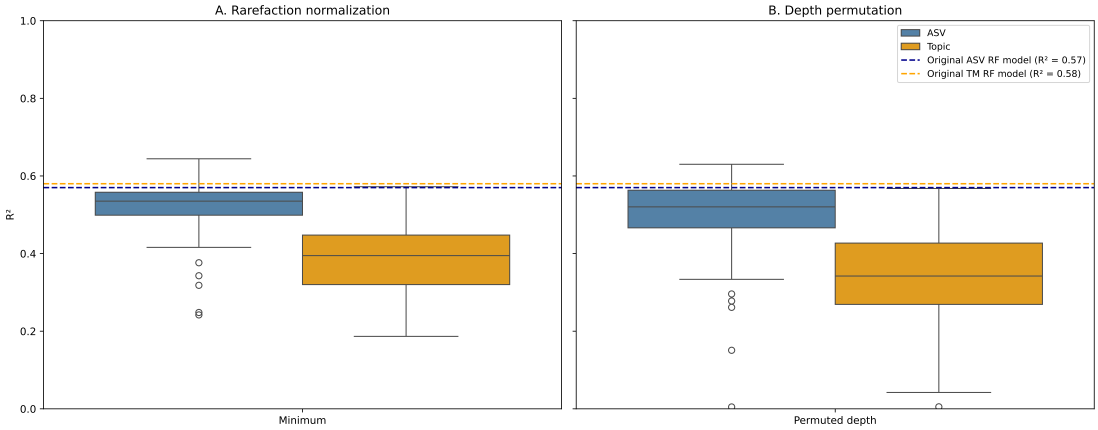

Fig. 14. The results of the data augmentation experiments. **A** Rarefaction approach to the minimum sequencing depth. **B** Permutation approach.

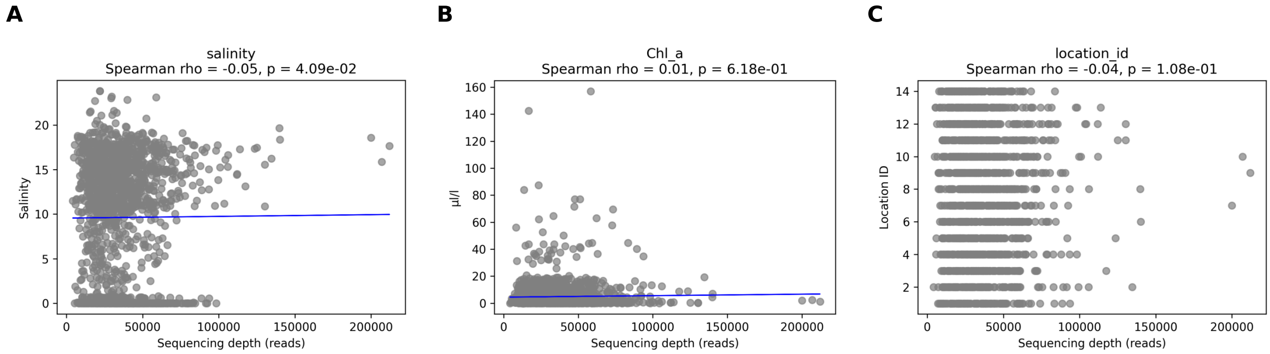

Fig. 15. The relationship between sequencing depth per sample and **A** salinity, **B** chlorophyll a and **C** location ID. For the numeric target variables, the fitted linear regression line is shown in blue. Values are widely scattered, and no clear trend is apparent. The low rho confirms that the sequencing depth explains very little of the observed variation. The regression lines are nearly flat, indicating no linear relationship between these variables and sequencing depth.

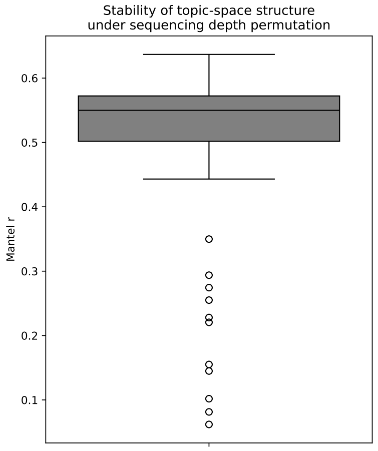

Fig. 16. Distribution of Mantel correlation coefficients (Mantel r) comparing sample-to-sample distance matrices derived from NNMF topic models of sequencing-depth-permuted datasets to the original sample-by-topic matrix.
